# HLA Ligand Atlas DIA: extending the benign immunopeptidomics resource with increased sensitivity through data-independent acquisition mass spectrometry

**DOI:** 10.1136/jitc-2025-012083

**Published:** 2025-08-31

**Authors:** Leon Bichmann, Ana Marcu, Daniel Johannes Kowalewski, Lena Katharina Freudenmann, Linus Backert, Lena Mühlenbruch, Maren Lübke, Philipp Wagner, Tobias Engler, Sabine Matovina, Mathias Hauri-Hohl, Roland Martin, Holger Moch, Luca Regli, Michael Weller, Markus W Löffler, Juliane S Walz, Oliver Kohlbacher, Hannes Röst, Hans-Georg Rammensee, Marian C Neidert

**Affiliations:** 1 Center for Systems and Engineering Immunology, Yale University, New Haven, Connecticut, USA; 2 Interfaculty Institute for Cell Biology and Immunology, University of Tübingen, Tübingen, Germany; 3 Genentech, San Francisco, California, USA; 4 Department of Peptide-based Immunotherapy, University Hospital Tübingen, Baden-Württemberg, Germany; 5 Obstetrics and Gynecology, University Hospital of Tübingen, University of Tübingen, Tübingen, Germany; 6 Pediatric Stem Cell Transplantation, University Children’s Hospital Zurich, University Hospital Zurich, Zurich, Switzerland; 7 Neuroimmunology and MS Research, Institute of Experimental Immunology, University of Zurich, Zürich, ZH, Switzerland; 8 Pathology, University Hospital Zurich, Zurich, Switzerland; 9 Clinical Neuroscience Center and Department of Neurosurgery, University Hospital Zurich, Zurich, Switzerland; 10 Neuroscience Center Zurich (ZNZ), University of Zurich, Zürich, Switzerland; 11 Laboratory of Molecular Neuro-Oncology, Department of Neurology, University Hospital Zurich, Zürich, Switzerland; 12 Institute for Clinical and Experimental Transfusion Medicine, University Hospital Tübingen, Tübingen, Germany; 13 Applied Bioinformatics, University of Tübingen, Tübingen, Germany; 14 Institute for Bioinformatics and Medical Informatics, University Hospitals Tubingen, Tübingen, Germany; 15 Donnelly Centre for Cellular and Biomolecular Research, University of Toronto, Toronto, Ontario, Canada; 16 Department of Neurosurgery, Cantonal Hospital St.Gallen, HOCH Health Ostschweiz, St. Gallen, SG, Switzerland

**Keywords:** Human leukocyte antigen - HLA, Major histocompatibility complex - MHC, Immunotherapy, Autoimmune, Infection

## Abstract

The human leukocyte antigen (HLA)-presented peptide repertoire, termed immunopeptidome, plays a crucial role for T-cell mediated immune reactions. Previously, the human immunopeptidome of non-malignant tissues has been mapped in a large-scale study, the HLA Ligand Atlas, via high-resolution data-dependent acquisition (DDA) mass spectrometry. This publicly available and user-friendly web interface (https://hla-ligand-atlas.org) is frequently used as a benign tissue reference in antigen discovery, especially for immunotherapy of cancer. Here, we extend the HLA Ligand Atlas resource with paired data-independent acquisition (DIA) runs for all tissue-subject combinations. This novel dataset comprises 946 DIA HLA class I and II immunopeptidomic runs from 242 non-malignant human samples across 18 subjects and 29 distinct tissues. Together with the published DDA runs, this extends the range and depth of analyses performed on the HLA Ligand Atlas dataset. In a concise analysis, we showcase advantages of DIA over DDA concerning spectral sampling and sensitivity. These findings are attributed to the increased dynamic range in DIA, enabling the identification of peptide transitions with low signal intensities. Moreover, we demonstrate the superior sensitivity by applying an HLA-A*02:01 allotype-specific spectral library search to identify and quantify HLA-presented peptides. We encourage reanalysis of the provided DDA and DIA data in combination as a reference for future research concerning human immunology.

## Background and summary

 The immunopeptidome comprises all peptides presented by the human leukocyte antigen (HLA) class I and class II molecules. The HLA-peptide complex presents a snapshot of the intracellular peptide repertoire to circulating T-lymphocytes. These can recognize HLA ligands originating from intracellular pathogens or cellular abnormalities. Understanding the repertoire of HLA-I and HLA-II ligands, their quantities and their variation across tissues and individuals in healthy and diseased states is crucial to develop T-cell-based immunotherapies for a broad range of diseases. The immunopeptidome can be exploited to develop T-cell-based immunotherapies in the context of infectious disease^1^,^3^ and cancer.^4^,^6^ The HLA-I and II immunopeptidomes also play an essential role in autoimmune diseases^7^ and transplant immunology.^8 9^ Thus, a deeper understanding of the ground-state immunopeptidome will advance our approach to define safe targets for T-cell-based immunotherapies.

Two previous studies initiated the systematic mapping of the normal-state immunopeptidome in multiple tissues in C57BL/6 mice and 21 humans^10^ using liquid chromatography coupled to tandem mass spectrometry (LC-MS/MS). The HLA Ligand Atlas dataset has been primarily used as a benign surrogate when defining tumor-associated antigens and encompasses 1274 LC-MS/MS runs in the well-established data-dependent acquisition (DDA) mode also called shotgun MS.

Data-independent acquisition (DIA) is a promising strategy for immunopeptidomics analysis, as it does not suffer from stochastic intensity-based precursor selection and instead measures all precursor ions including low-intensity ones which results in higher sensitivity. On the other hand DIA spectra are more complex to analyze as they are often highly multiplexed by several peptide species and require deconvolution for instance by the use of spectral libraries—a collection of high-quality peptide-spectrum matches derived from comparable DDA measurements. Recently also library-free approaches for instance through in silico predicted spectral libraries for any peptide sequence have become more accurate,^11^,^13^ nevertheless sample-specific library-based analysis is still the most reliable approach to analyze DIA data as it does not suffer from prediction inaccuracies. Ultimately, HLA allele-specific spectral libraries whether generated sample-specific or retrieved from public repositories such as for instance the SysteMHCAtlas^14^ allow limiting the inflated unspecific cleavage search space faced in immunopeptidomics DDA database search approaches,^15^,^19^ given the HLA allele of interest is well-studied and contained in the respective repository.

Here we extend the established HLA Ligand Atlas data resource with 946 matched LC-MS/MS runs acquired in DIA mode on the exact same Orbitrap Fusion Lumos mass spectrometer. Two replicate DIA runs were acquired for the same samples and subjects covered in DDA mode in the HLA Ligand Atlas resource, which makes it possible to carry out classical library-based DIA analyses. All data is well curated and labeled in correspondence to the previously published DDA data. It is publicly available in the Proteomics Identification Database (PRIDE) online repository.^20^

While comparing DDA and DIA processing results in general, we also demonstrate the use of patient and HLA allele-specific libraries combining peptide identifications from multiple DDA LC-MS/MS runs. As a result, we demonstrate that using DIA for immunopeptidomics resulted in an approximate twofold reduction of missing values in peptide identifications across different tissues and individuals improving the comparability across them. Thus by extending the HLA Ligand Atlas resource with matched DIA data we provide a way for researchers to compare their data to the benign surrogate reference with increased sensitivity for instance to define tumor-exclusive antigens and others.

## Methods

### Human tissue samples

Tissue samples from different organs were obtained during autopsy or surgery. An autopsy was performed no later than 72 hours *postmortem*. Tissue samples were immediately snap-frozen in liquid nitrogen. None of the subjects included in this study suffered from malignant disease. Tissue and organ annotation was performed by a board-certified pathologist and their use for scientific research approved by the corresponding ethics committee as described in detail previously.^10^

Details on all samples are described further in the original HLA Ligand Atlas study illustrating the data analysis obtained by DDA acquisition.^10^

### HLA immunoaffinity purification

HLA ligands were eluted by standard immunoaffinity chromatography as described in the original HLA Ligand Atlas study.^10^ Briefly, tissues were minced in lysis buffer consisting of CHAPS (PanReac AppliChem, Darmstadt Germany) and one cOmplete protease inhibitor cocktail tablet (Roche, Basel, Switzerland) in Phosphate-buffered saline. The pan HLA class I antibody W6/32^21^ and the pan HLA class II antibody Tü39 and the HLA-DR specific antibody L243 were generated in house. More details on the immunoaffinity purification can be found in online supplemental information S3.

### JY cell culture and isolation of HLA ligands

The JY cell line (ECACC 94022533, IHW9287) is an Epstein-Barr virus-immortalized B cell lymphoblastoid line, with the advantageous property of being homozygous for all HLA-I loci (HLA-A*02:01, HLA-B*07:02, and HLA-C*07:02). The JY HLA-I ligandome was isolated through immunoaffinity purification. More details on the JY cell culturing can be found in online supplemental information S4.

### Serial dilution of JY eluate and synthetic peptide spike-in

The purified JY HLA-I peptide solution was spiked with a cocktail of 10 heavy isotope labeled synthetic peptides diluted to a stock solution of 10 fmol/µL. The synthetic peptides spiked into sample No. 1 are described in detail in online supplemental information table S1. 10 different tryptic peptides covering the retention time range of HLA ligands were selected and synthesized with an L+7 heavy isotopic modification to allow easy distinction between native and spiked-in peptides. The synthetic peptide mix, having a stock concentration of 10 fmol/µL, was diluted with the JY HLA-I peptide eluate to 0.2 fmol/µL for each synthetic peptide.

The JY peptide eluate spiked with 0.2 fmol/µL of each synthetic peptide was named sample No. 1 and was diluted 1:2 with 1% ACN/0.05% TFA resulting in sample No. 2, which in turn was diluted 1:2 with 1% ACN/0.05% TFA to obtain sample No. 3, etc. This process resulted in five serial dilutions that were aliquoted and stored at −80°C until LC-MS/MS analysis (table 1).

**Table 1:** The HLA ligand solution isolated from the JY cell line was spiked with synthetic peptides resulting in sample No. 1, which was subsequently serially diluted up to sample No. 5

Dilution factor	Sample	Final concentration per synthetic peptide
1	No. 1	0.2 fmol/µL
1:2	No. 2	0.1 fmol/µL
1:4	No. 3	0.05 fmol/µL
1:8	No. 4	0.025 fmol/µL
1:16	No. 5	0.0125 fmol/µL

ACN, Acetonitrile; DN, subject (organ donor); FDR, False discovery rate; HLA, human leukocyte antigen; LFQ, label-free quantification; OVA, Ovary; PRIDE, Proteomics Identification Database; TFA, Trifluoroacetic acid; THY, Thymus; XIC, Extracted-ion chromatogram.

### LC-MS/MS data acquisition

HLA ligands were characterized by LC-MS/MS employing an Ultimate 3000 RSLC Nano UHPLC System (Thermo Fisher Scientific, San Jose, California, USA) coupled on-line to an Orbitrap Fusion Lumos mass spectrometer (Thermo Fisher Scientific) equipped with a Nanospray Flex Ion Source (Thermo Fisher Scientific). Peptides were trapped on a 75 µm × 2 cm Acclaim PepMap 100 C18 Nanotrap column (Thermo Fisher Scientific) with 1% ACN/0.05% TFA at a flow rate of 4 µL/min for 10 min. Peptides were separated on a 50 µm x 25 cm PepMap RSLC C18 column (Thermo Fisher Scientific), with a particle size of 2 µm. Peptides were eluted with a linear gradient from 3% to 40% solvent B (80% ACN, 0.15% FA in water) at a flow rate of 0.3 µL/min over 90 min.

MS data acquisition was optimized for HLA-I and HLA-II ligands. DDA methods and associated data were thoroughly described and published in our previous study^10^ and the same Orbitrap Fusion Lumos instrument as well as similar scan mass ranges for fragmentation were employed (DIA-HLAI and DDA-HLAI: 400–650 m/z, DIA-HLAII: 450–950 m/z, DDA-HLAII-400–1000 m/z). DIA methods are described in detail in online supplemental information S2.

### Computational LC-MS data processing

#### Spectral library generation

Spectral libraries were generated from matching DDA runs and peptide identifications at 1% peptide-level FDR in the HLA Ligand Atlas PRIDE repository (PRIDE accession PXD020186) using the nf-core/diaproteomics workflow^22^ V.1.2.2. The workflow makes use of the software EasyPQP (https://github.com/grosenberger/easypqp) to annotate transitions of peptide identifications and their intensities retrieved from corresponding DDA raw data as spectral library. To create aligned spectral libraries of individual DDA replicates into the same retention time (RT) space, a pairwise linear RT alignment is carried out between libraries. Only spectra with at least four transitions and a maximum of 6 transitions out of all were included.

Dynamic range libraries: DDA MS runs were processed using the nf-core/mhcquant workflow^23^ V.1.6.0 using default settings and applying the isotope labels from online supplemental table S1 as variable modification. As for the technical replicate sampling benchmark peptide identifications of all three DDA replicates of the first dilution step were used to create the spectral library. For the comparison across dilution steps the library was created combining all peptide identifications of the 15 DDA MS runs of the dilution series (PRIDE accession PXD024809).

Pan-subject library AUT-DN12: As for the replicate benchmark for AUT-DN12 spleen the library was created combining all peptide identifications from the three DDA technical replicates of the spleen sample of autopsy subject AUT-DN12. For the cross-tissue comparison all peptide identifications from all 48 DDA MS runs of subject AUT-DN12 were combined into a library (the exact file list is provided in the PRIDE repository (accession PXD024837) as HLA_Atlas_DDA_DN12_sample_sheet.tsv).

Pan-allele library A*02:01: The library was created combining peptide identifications predicted to bind to HLA-A*02:01 from 153 DDA MS runs of the subjects AUT-DN04, AUT-DN12, AUT-DN14 and AUT-DN15 (the exact file list is provided in the PRIDE repository (PRIDE accession PXD024837) as HLA_Atlas_DDA_A02_sample_sheet.tsv).

### DIA data processing

DIA MS runs were processed using the nf-core/diaproteomics workflow V.1.2.2 (https://github.com/nf-core/diaproteomics). The workflow integrates the OpenSwathWorkflow^24^ for targeted extraction, Pyprophet^25^ for FDR scoring and DIAlignR^26^ for chromatogram alignment. A peptide-level FDR cut-off was kept at 0.01 using ms1 and ms2 information.

Dynamic range experiment: The 10 HLA-I DIA MS runs of the JY cell dilution series (described above from the PRIDE repository, PRIDE accession PXD024809) were processed applying the corresponding dilution series master library.

Pan-subject analysis AUT-DN12: 16 HLA-I DIA MS runs of the tissues: spleen, lung, kidney, bone marrow, small intestine, liver, lymph node and ovary from the subject AUT-DN12 (the exact file list is provided in the PRIDE repository (PRIDE accession PXD024837) as HLA_Atlas_DIA_DN12_sample_sheet.tsv) were processed applying the corresponding pan-subject master library.

Pan-allele analysis A*02:01: 50 HLA-I DIA MS runs of the tissues: spleen, lung, kidney, bone marrow, liver, lymph node, brain and ovary from the subjects AUT-DN04, AUT-DN12, AUT-DN14 and AUT-DN15 (the exact file list is provided in the PRIDE repository (PRIDE accession PXD024837) as HLA_Atlas_DIA_A02_sample_sheet.tsv) were processed applying the corresponding pan-allele master library.

### Hierarchical clustering of tissues according to peptide quantities

The matrix of peptide quantities across samples resulting from the DIA data processing using the pan-HLA-A*02:01 specific spectral library was used for the hierarchical clustering approach. Only the four patients (AUT-DN04, AUT-DN12, AUT-DN14 and AUT-DN15) sharing the allele HLA-A*02:01 and among those, only tissues that are shared by at least two individuals were considered for the approach. Only peptide identifications that were retrieved by at least one sample of each subject were considered. The clustering was carried out with the Python package seaborn V.0.8.1 using Euclidean distance and average linking as cluster parametrization.

## Results

### Spectral sampling and sensitivity of DDA versus DIA as illustrated by a JY dilution experiment

In order to demonstrate the differences in spectral sampling and sensitivity between the DDA and DIA methodology employed for the here described data sets, we investigated the influence of the acquisition method in a JY cell line immunopeptidome dilution series using the identical assay and experimental design as employed for the HLA Ligand Atlas samples. For this the JY cell line immunopeptidome extract was diluted at the ratio 1:10 in five serial steps and further analyzed.

**Figure 1:**
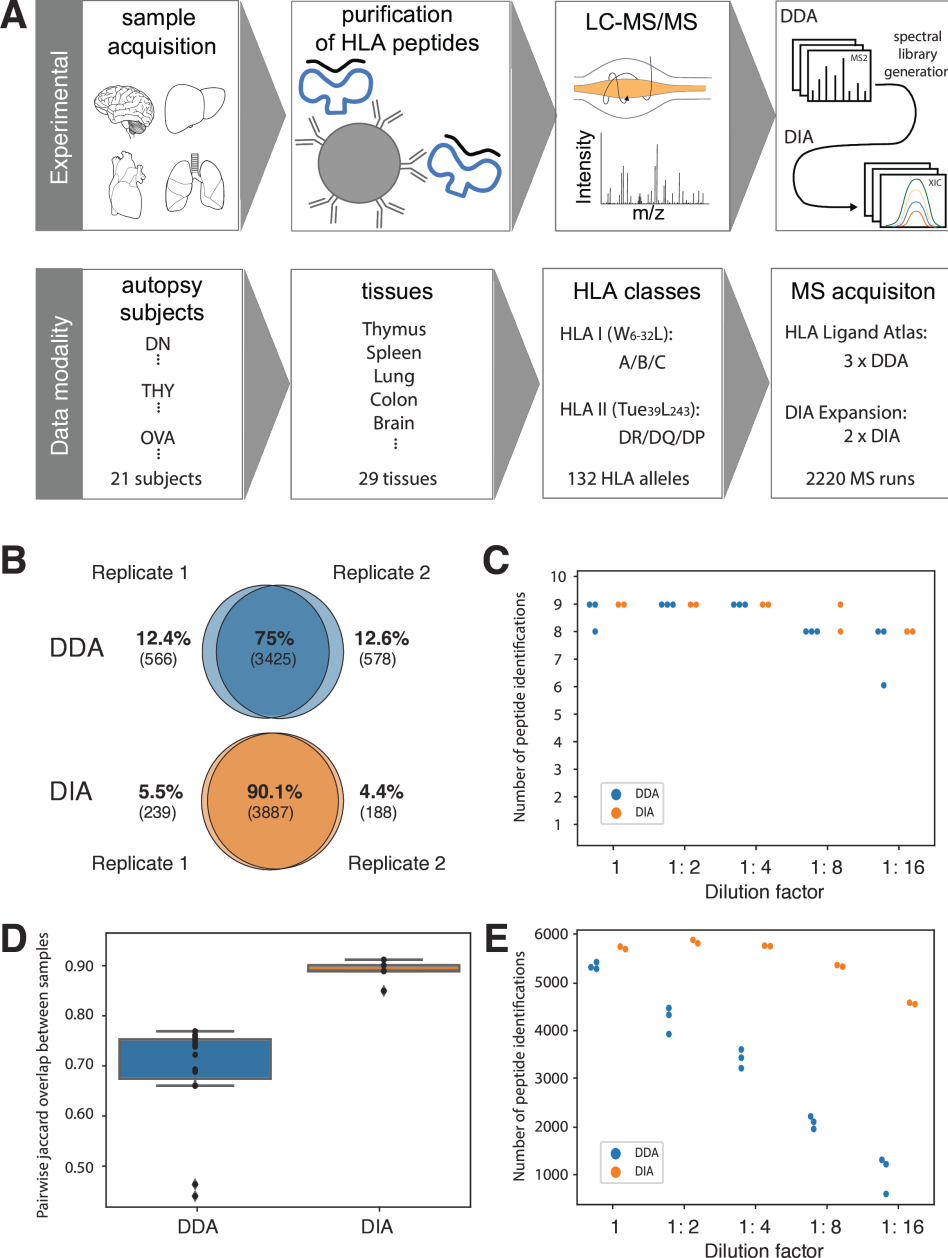
(**A**) Scheme illustrating the experimental design and data acquisition strategy for the HLA ligand atlas data across subjects. Panels B–E describe the comparative analysis of DDA and DIA data obtained from the dynamic range experiment based on HLA-I peptide extracts from the JY cell line. (**B**) Peptide identification overlap across two replicates of the first dilution step. (**C**) The number of spiked-in isotope-labeled peptides found at each dilution step. (**D**) Pairwise Jaccard overlaps across replicates for each dilution step. (**E**) The number of JY cell line background HLA-bound peptides found at each dilution step. DDA, data-dependent acquisition; DIA, data-independent acquisition; HLA, human leukocyte antigen; LC-MS/MS, liquid chromatography coupled to tandem mass spectrometry.

Already at the highest concentration a clear difference in spectral sampling between DDA and DIA is evident. The consensus of peptide identifications between two technical replicate measurements is above 90% for the DIA approach, in contrast to 75% for the DDA approach (figure 1B). Comparing the pairwise Jaccard overlap between all technical replicates at all dilution steps yields a similar result of an interquartile value range equal to 0.9–0.91 for the DIA approach and 0.73–0.76 for the DDA approach (figure 1C).

Finally, the difference in sensitivity between DDA and DIA approaches was assessed by the count of identified peptides at each dilution step. While DDA indicates a linear trend of less peptide identifications at higher dilution rates, DIA can retrieve similar numbers of peptides at a much slighter decrease towards lower concentrations (figure 1D). This confirms the increased dynamic range of the DIA approach, enabling more sensitive peptide detection of the low intensity range.

### Complementary aspects and comparison of missing values for DDA and DIA in the HLA Ligand Atlas datasets

**Figure 2:**
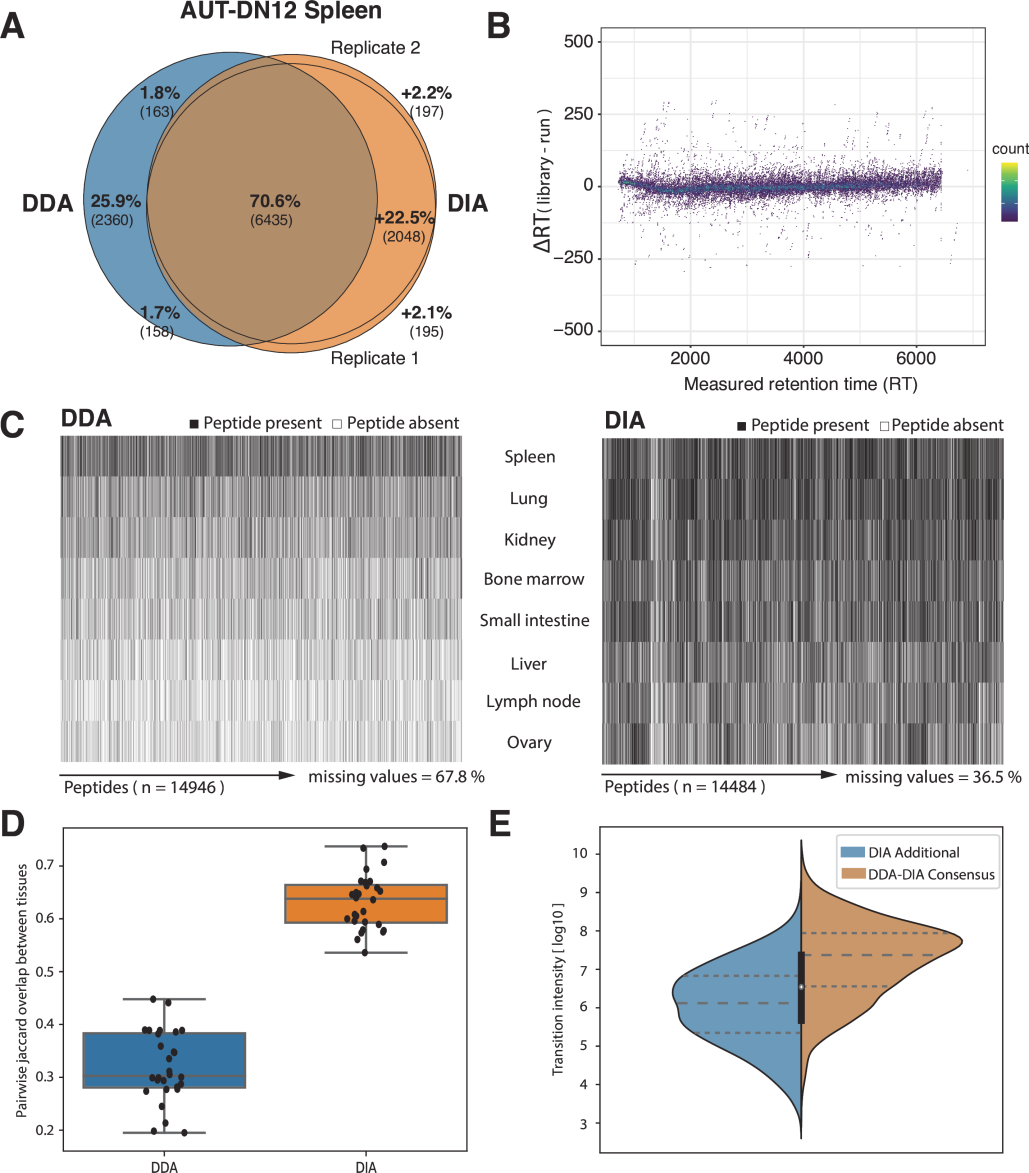
Panels A–E illustrate the difference in peptide identifications and their properties across tissue samples of subject AUT-DN12 using DDA and DIA. For all panels in figure 2, we searched individual DIA runs with an AUT-DN12 pan-tissue spectral library. (**A**) Peptide identification overlap between the union of three DDA and two DIA runs as exemplified by AUT-DN12 spleen. (**B**) The retention time difference between the library and the experiment of all DIA-retrieved peptides across the entire RT range. (**C**) Missing values across multiple tissues in the peptide identification data matrix (entries are black if a peptide is present or white if absent). (**D**) Pairwise Jaccard overlaps of the peptide identification matrix across multiple tissues. (**E**) Comparative distribution of transition intensities of peptides retrieved in consensus by DDA and DIA (orange) compared with peptides identified in DIA runs alone (blue). DDA, data-dependent acquisition; DIA, data-independent acquisition.

The current strategy to analyze immunopeptidomics data obtained via DIA acquisition is to generate a spectral library that best describes the expected peptide fragmentation patterns. In the case of the HLA Ligand Atlas, we relied on DDA data to generate a pan-tissue library for each subject. We generated a pan-subject spectral library by combining 48 DDA LC-MS/MS runs of multiple tissues of the AUT-DN12 autopsy subject. When analyzing the HLA-I immunopeptidome of AUT-DN12 spleen tissue with the pan-tissue AUT-DN12 library, we were able to confirm about 70% of the previously detected peptides via both DIA and DDA approaches (figure 2A). In addition, we found about ∼2,000 peptides (26%) exclusively identified via either the DDA or the DIA approach (figure 2A). The additional peptides that could be identified on the spleen using the DIA approach were previously identified exclusively on other tissues, indicating an advantage to increasing the library search space across one subject. In addition, the retention time of peptides identified in DIA mode broadly followed the expected trends, with only slight variations between the DDA data represented in the spectral library and the empirical retention time in DIA mode, further supporting the confidence of the DIA-based identifications (figure 2B). Uniquely identified peptides using the DDA approach might be due to differences in the DIA spectral library inclusion filter criteria and the downstream scoring of the DIA approach.

Next, we searched a multi-tissue set of subject AUT-DN12 HLA-I immunopeptidomics DIA LC-MS/MS runs, obtained from analyzing the spleen, liver, lung, kidney etc, with the pan-subject spectral library and observed an increase in the number of identified peptides and a reduction of missing values across the different tissue samples (figure 2C). When dissecting the share of peptides across various tissues, the DIA approach results in a more complete data matrix than the DDA approach reducing the number of missing values by nearly half from 67.8% to 36.5% (figure 2C). This is reflected by a higher range of pairwise Jaccard overlaps across the tissues at an interquartile value range of 0.28–0.38 for DDA and 0.59–0.66 for DIA (figure 2D). Moreover, the intensity range of additional peptide identifications across tissues that were retrieved exclusively using DIA, adheres to a distribution that is in median one order of magnitude lower (median intensity=1.3×10^6^) than those identified in consensus (median intensity=2.2×10^7^) by DDA and DIA (figure 2E). This highlights the increased sensitivity and dynamic range of the DIA data acquisition method. We can recapitulate this observation when looking at individual peptide transition intensities across DDA and DIA replicates of the same sample (online supplemental figure S1).

### Application of a pan-HLA-A*02:01 spectral library across tissues and subjects

**Figure 3:**
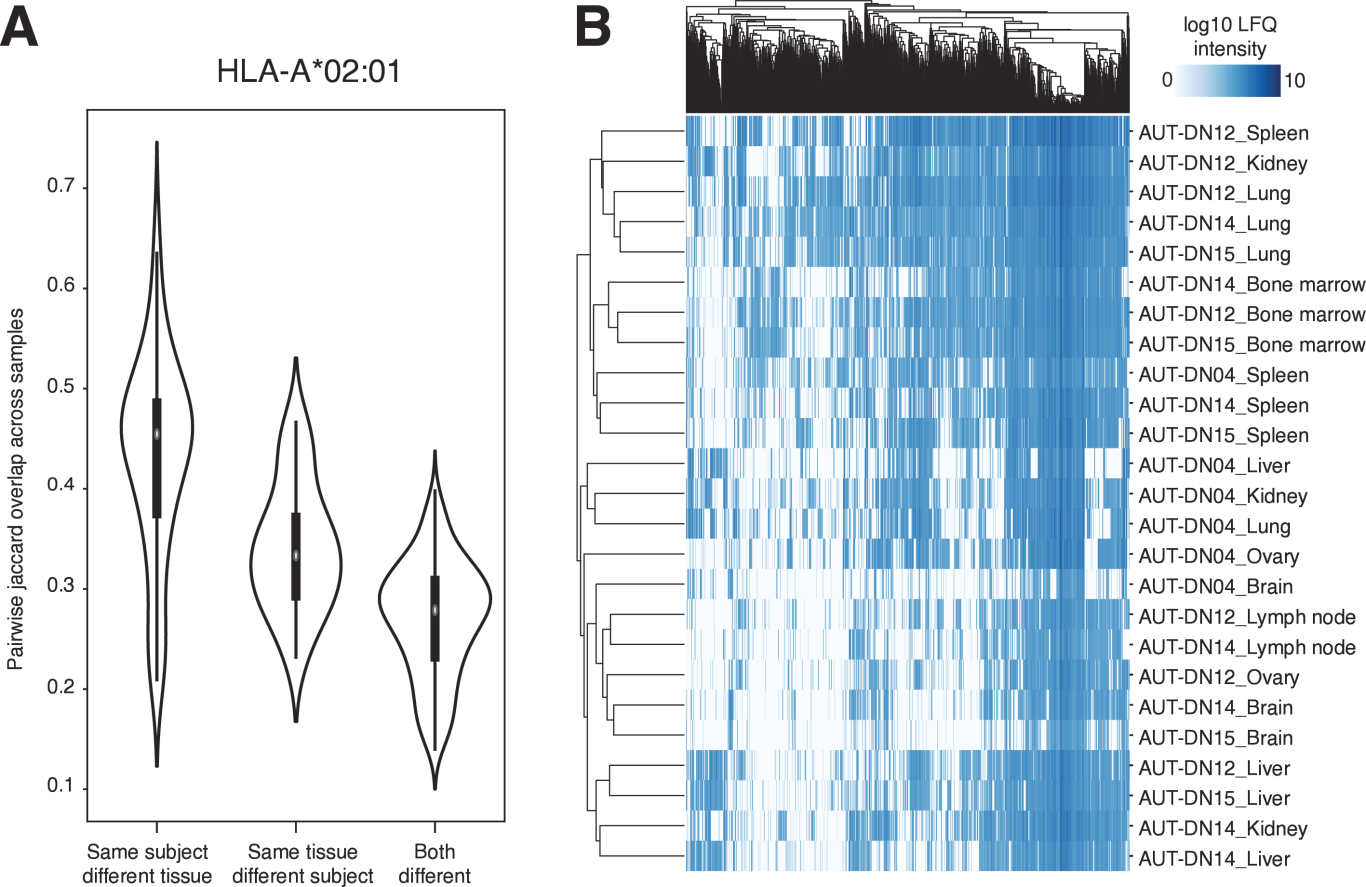
Results of the application of a pan-HLA-A*02:01 based spectral library to DIA measured samples of multiple subjects and tissues. (**A**) Modes of variability shown through distribution of pairwise Jaccard overlaps differentiating between samples of (1) same subjects but different tissues, (2) same tissues but different subjects or (3) both different. (**B**) Quantitative peptide identification matrix (annotated with label-free quantification (LFQ) log10 intensity) after filtering for peptides present in at least one sample of every subject. The matrix was sorted by hierarchical clustering and reveals immunopeptidome-level similarities of tissues from different subjects. DIA, data-independent acquisition; HLA, human leukocyte antigen.

Comparing the immunopeptidome of multiple individuals is hindered by the fact that it is highly personalized due to the individual HLA types of each subject and in order to apply the DIA approach across multiple subjects, a different strategy is to tune the used spectral library to the common set of shared HLA alleles. This strategy incorporates intersubject variability, while maintaining HLA-allotype specificity. Previous studies based on DDA data showed that variability is higher between subjects than between different tissues.^10^ Hence, we sought to investigate whether the use of pan-subject HLA-specific libraries would enhance the comparability of immunopeptidomes across multiple subjects and therefore reduce subject-specific signals. Therefore, we generated a pan-HLA-A*02:01 spectral library from 153 DDA LC-MS/M runs from various tissues and subjects carrying this allele (AUT-DN04, AUT-DN12, AUT-DN14 and AUT-DN15). The peptides included in the HLA-A*02:01 library were predicted to bind to this allotype. By employing an HLA-A*02:01 specific library, we were able to confirm the observations obtained from the DDA HLA-I immunopeptidome data, in that different tissues from the same subject were most similar to each other, as opposed to the same tissue across subjects (figure 3A).

Therefore, we conclude that despite the increased sensitivity of the DIA data acquisition approach, the highest variability is observed across subjects even when controlling for the HLA allotype. However, when artificially eliminating subject-specific effects by focusing on peptides that are found at least in one tissue of every subject in this comparison, shared tissue-specific effects become evident (figure 3B). Clustering the reduced matrix of peptides and their quantities reveals higher similarities of the same tissues across multiple subjects than various tissues of the same subject, which is in concordance with previous and novel findings of tissue-specific patterns of HLA presentation.^10 27^

## Conclusion

In conclusion, we expand the HLA Ligand Atlas DDA data with paired DIA data. Our analysis demonstrates how the novel DIA HLA Ligand data can be analyzed based on spectral libraries generated from our previously published DDA dataset. Pan-HLA spectral libraries can be applied to multiple subjects and tissues in the HLA Ligand Atlas data to achieve greater sensitivity and similar conclusions can be drawn from the DDA and DIA analysis. Ultimately, the results validate the overall quality and comparability of the two data sets, and we encourage further reanalysis of the human immunopeptidome in this regard.

## Data availability

All associated data for this manuscript has been deposited in the Proteomics Identification Database (PRIDE). The DDA runs of the HLA Ligand Atlas project can be found in the PRIDE repository (PRIDE accession PXD020186).^28^ DIA runs of the HLA Ligand Atlas project can be found in the PRIDE repository (PRIDE accession PXD024837).^29^ DDA and DIA runs of the JY Dilution experiment can be found in the PRIDE repository (PRIDE accession PXD024809).^30^ Spectral libraries and pseudo-IRTs are stored as txt and search results as csv files in the corresponding repositories.

## Code availability

The open-source data analysis workflows that were employed for this study are provided for free as part of the GitHub repositories of the nf-core initiative for reproducible bioinformatics pipelines:

nf-core/diaproteomics V.1.2.2:

https://github.com/nf-core/diaproteomics

nf-core/mhcquant V.1.6.0:

https://github.com/nf-core/mhcquant

## Supplementary material

10.1136/jitc-2025-012083 online supplemental file 1

## Data Availability

Data are available in a public, open access repository.
