# Supplementary material for: HLA Ligand Atlas DIA: extending the benign immunopeptidomics resource with increased sensitivity through data-independent acquisition mass spectrometry
Source: J Immunother Cancer. 2025 Aug 31;13(8):e012083. doi: 10.1136/jitc-2025-012083 (PMC12406853; doi:10.1136/jitc-2025-012083)
Supplement: online supplemental file 1 [file jitc-13-8-s001.docx]

SUPPLEMENTARY INFORMATION

**Figure S1: Examples XICs of peptide identifications recovered using the DIA approach**


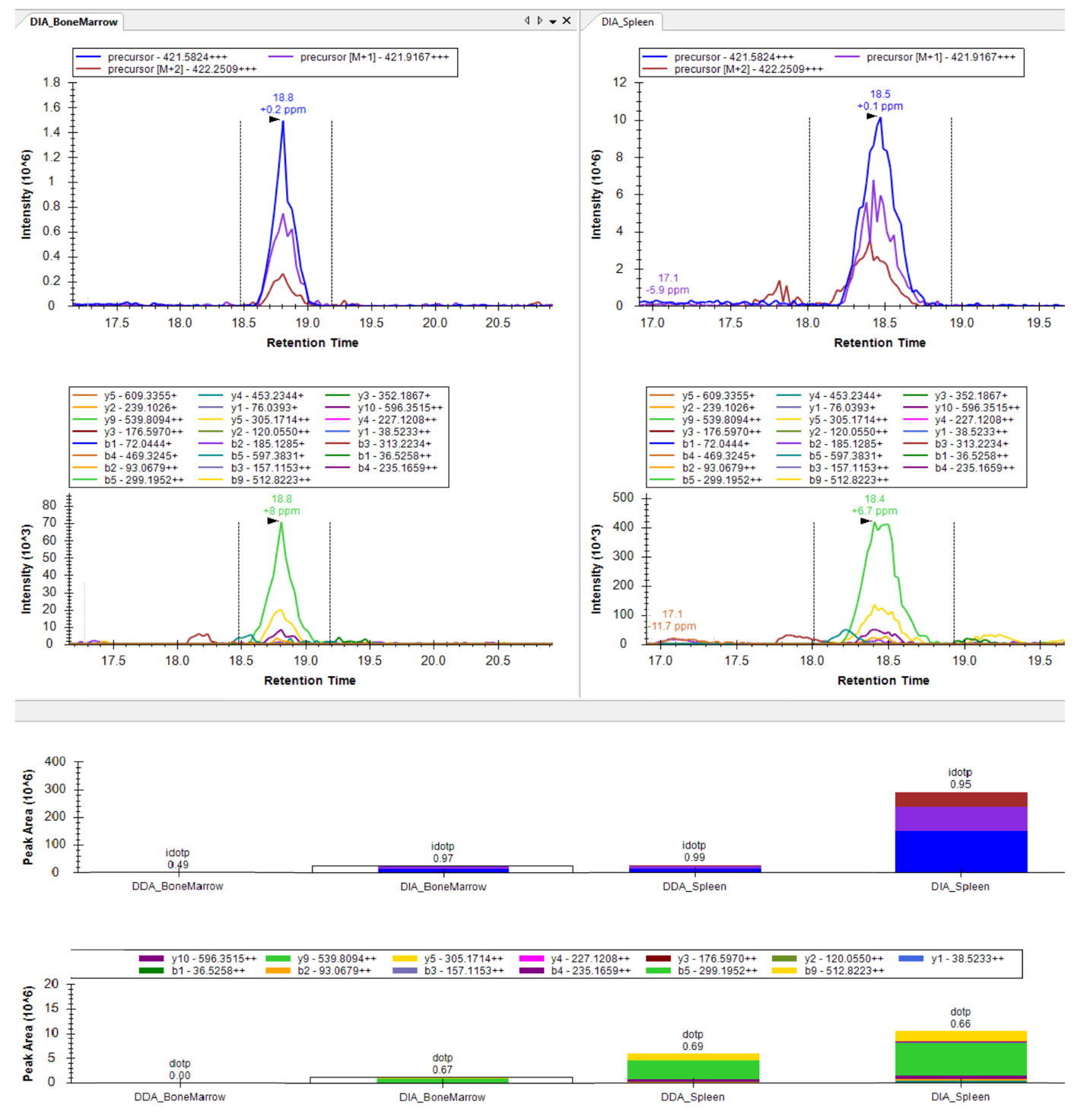


Figure S1: *Examplary XICs of the same peptide identification recovered in bone marrow and spleen tissue using the DIA approach in contrast to the DDA approach visualized through Skyline. Transition quantities are low in some of the DDA runs, which might have lead to the missing discovery in these samples. In contrast DIA recovers the peptide transition in all MS runs.*

**Table S1: Serial Dilution of JY Eluate and Synthetic Peptide Spike-In**

Table S1: Heavy isotope-labeled peptides that were spiked into sample No.1 consisting of JY HLA ligand eluate. The final concentration per synthetic peptide in sample No.1 was 0.2 fmol/µl.

| Peptide | Modification (L+7) | Spike-in concentration (No.1) |
| --- | --- | --- |
| ISLGEHEGGGK | IS[L(^13^C6; ^15^N)]GEHEGGGK | 0.2 fmol/µl |
| VGASTGYSGLK | VGASTGYSG[L(^13^C6; ^15^N)]K | 0.2 fmol/µl |
| TLIAYDDSSTK | T[L(^13^C6; ^15^N)]IAYDDSSTK | 0.2 fmol/µl |
| FLASSEGGFTK | F[L(^13^C6; ^15^N)]ASSEGGFTK | 0.2 fmol/µl |
| GFLDYESTGAK | GF[L(^13^C6; ^15^N)]DYESTGAK | 0.2 fmol/µl |
| ALFSSITDSEK | A[L(^13^C6; ^15^N)]FSSITDSEK | 0.2 fmol/µl |
| HFALFSTDVTK | HFA[L(^13^C6; ^15^N)]FSTDVTK | 0.2 fmol/µl |
| VYAETLSGFIK | VYAET[L(^13^C6; ^15^N)]SGFIK | 0.2 fmol/µl |
| GASDFLSFAVK | GASDF[L(^13^C6; ^15^N)]SFAVK | 0.2 fmol/µl |
| FFLTGTSIFVK | FF[L(^13^C6; ^15^N)]TGTSIFVK | 0.2 fmol/µl |

**S2: LC-MS/MS Data Acquisition**

DIA methods consisted of full scans acquired as tSIM spectra in profile mode at a resolution of 120,000 at m/z 200 with a target value of 1.5e5. DIA isolation windows were adjusted to precursor density resulting in two different non-overlapping isolation window widths that varied for HLA-I and HLA-II, resulting in DIA1 and DIA2 experiments. Spectra were acquired in Top20 mode for both tSIM scans, DIA1 and DIA2 experiments with 100 ms maximum injection time. Similar to the DDA acquisition method, we limited the precursor mass range to accommodate HLA-I and HLA-II ligand length distribution.

HLA-I ligands were fragmented with CID at a collision energy of 35% and an activation Q of 0.25. The Orbitrap resolution was set to 30,000. The tSIM scan isolation was performed in the quadrupole with an isolation width of m/z 252 centered at m/z 525. Thereby, we obtained isolation windows ranging from m/z 399 – 651. Mass windows were applied as follows: The mass window for the DIA1 experiment was set to 15 m/z, resulting in 10 DIA windows in covering an interval from 400 m/z - 550 m/z. The DIA2 experiment consisted of 34 m/z mass windows, resulting in 3 DIA windows covering 550 m/z - 650 m/z.

HLA-II ligands were fragmented with HCD with collision energy set to 30%. The Orbitrap resolution was set to 30,000. The tSIM isolation was centered at m/z 700 with an isolation width of m/z 502 resulting in a mass range of 449 to m/z 951. The following mass windows were applied: DIA1 isolation windows were 25 m/z wide, resulting in 10 DIA windows covering 450 m/z - 700 m/z. DIA2 experiments consisted of 36 m/z isolation windows, resulting in 7 DIA windows covering 700 m/z - 950 m/z.

Table S3: A detailed overview of the applied mass windows applied in the DIA method:

| **HLA-I** | | | | | **HLA-II** | | | | |
| --- | --- | --- | --- | --- | --- | --- | --- | --- | --- |
| **Experiment** | **Mass List Table m/z** | **Isolation width m/z** | **Start m/z** | **End m/z** | **Experiment** | **Mass List Table m/z** | **Isolation width m/z** | **Start m/z** | **End m/z** |
| **tSIM** | 525 | 252 | 399 | 651 | **tSIM** | 700 | 502 | 449 | 951 |
| **DIA 1** | 407.95375 | 15 | 400.45 | 415.4575 | **DIA 1** | 462.98125 | 25 | 450.475 | 470.485 |
|  | 422.96125 | 15.0075 | 415.4575 | 430.465 |  | 487.99375 | 25.0125 | 475.4875 | 495.4975 |
|  | 437.96875 | 15.0075 | 430.465 | 445.4725 |  | 513.00625 | 25.0125 | 500.5 | 520.51 |
|  | 452.97625 | 15.0075 | 445.4725 | 460.48 |  | 538.01875 | 25.0125 | 525.5125 | 545.5225 |
|  | 467.98375 | 15.0075 | 460.48 | 475.4875 |  | 563.03125 | 25.0125 | 550.525 | 570.535 |
|  | 482.99125 | 15.0075 | 475.4875 | 490.495 |  | 588.04375 | 25.0125 | 575.5375 | 595.5475 |
|  | 497.99875 | 15.0075 | 490.495 | 505.5025 |  | 613.05625 | 25.0125 | 600.55 | 620.56 |
|  | 513.00625 | 15.0075 | 505.5025 | 520.51 |  | 638.06875 | 25.0125 | 625.5625 | 645.5725 |
|  | 528.01375 | 15.0075 | 520.51 | 535.5175 |  | 663.08125 | 25.0125 | 650.575 | 670.585 |
|  | 543.02125 | 15.0075 | 535.5175 | 550.525 |  | 688.09375 | 25.0125 | 675.5875 | 695.5975 |
| **DIA 2** | 567.5335 | 34 | 550.5335 | 584.5335 | **DIA 2** | 718.609 | 36 | 700.609 | 736.609 |
|  | 601.5505 | 34.017 | 584.542 | 618.559 |  | 754.627 | 36.018 | 736.618 | 772.636 |
|  | 635.5675 | 34.017 | 618.559 | 652.576 |  | 790.645 | 36.018 | 772.636 | 808.654 |
|  |  |  |  |  |  | 826.663 | 36.018 | 808.654 | 844.672 |
|  |  |  |  |  |  | 862.681 | 36.018 | 844.672 | 880.69 |
|  |  |  |  |  |  | 898.699 | 36.018 | 880.69 | 916.708 |
|  |  |  |  |  |  | 934.717 | 36.018 | 916.708 | 952.726 |

**S3: HLA Immunoaffinity purification**

The antibodies were coupled to sepharose beads (Sigma-Aldrich, St. Louis, MO) according to the manufacturers’ protocol. For the isolation of HLA class II molecules equal amounts of Tü39 and L243 antibody were mixed. Immunoprecipitation was performed against HLA-I and HLA-II molecules by setting up the affinity columns in series, allowing the lysate to pass both columns cyclically overnight at 4°C. Affinity columns were washed for 30 minutes with PBS and for 1 h with water. Four elution steps were performed through incubation with 0.2% TFA. The eluted fractions were pooled. Eluted peptides were further purified by ultrafiltration employing 3 kDa and 10 kDa Amicon filter units (Merck Millipore, Darmstadt Germany) for HLA-I and HLA-II ligands respectively. The eluate volume was reduced by lyophilization or vacuum centrifugation to about 50 µl. Finally, the peptides were purified five times using ZipTip Pipette Tips with C18 resin and 0.6 µl bed volume (Merck) and eluted in 32.5% ACN/0.2% TFA. The purified peptide solution was concentrated by vacuum centrifugation and supplemented with 1% ACN/0.05% TFA and stored at −80°C until LC-MS/MS analysis.

**S4: JY Cell Culture and Isolation of HLA Ligands**

A frozen 1 ml JY cell suspension containing  cells in freezing medium consisting of 10% dimethyl sulfoxide (DMSO, WAK Chemie, Steinbach/ Taunus, Germany) in fetal calf serum (FCS, Capricorn Scientific, Ebersdorfergrund, Germany) was thawed by dilution with RPMI 1640 medium (Thermo Fisher Scientific) supplemented with 1% sodium pyruvate (100 mM) (Thermo Fisher Scientific, San Jose, CA), 1% MEM non-essential amino acids (100x) (Thermo Fisher Scientific), 1% Penicillin (103 x U/ml)/Streptomycin (10 g/ml) (Sigma-Aldrich, St. Louis, MO), and 10% FCS. Cells were cultured in humidified incubators at 37°C and 7.5% CO_2_. Cells were split every two to three days at a ratio of 1:2, 1:3 or 1:5, depending on cell proliferation and pH indicator color change in medium. For harvesting, culture medium was removed by centrifugation (1500 rpm, 15 min, room temperature, with break), and cells were washed twice with Dulbecco's Phosphate-Buffered Saline (Thermo Fisher Scientific) to remove residual medium. Pellets were pooled to obtain an approximate 5 ml pellet containing about 3 - 4 x 10^9^ cells and were frozen and stored at -80°C until immunopurification of HLA ligands was performed.

Deviations from the standard procedure were motivated by the large number of cells used. Briefly, a 7 ml cell pellet, approximately containing 4 x 10^9^ cells was employed as input material. The cell pellet was lysed in 30 ml 2x lysis buffer, and 10 ml 1x lysis buffer. Subsequently, the cell lysate was cleared by two consecutive centrifugation steps at maximum speed, and 4°C for 45 minutes. The cleared lysate was loaded onto Econo Columns with 5 cm diameter (Bio-RAD, Hercules, CA), containing 20 ml antibody-bead conjugates (1 mg/ml) and was run cyclically over-night. Peptides were eluted into 5 ml low-bind tubes (Eppendorf, Hamburg, Germany), the first elution being performed with 100 µl 10% TFA and 600 µl 0.2% TFA (followed by three subsequent elution steps with 600 µl 0.2% TFA). HLA-I ligands were further purified by ultrafiltration employing a 10 kD filter unit suitable for a 15 ml tube (Falcon, Corning, NY). Peptide solutions were lyophilized to complete dryness and resuspended in 3 ml 1% ACN/0.05% TFA.

**S5: Computation of Jaccard coefficients between samples**

We investigated the similarity between replicates, samples and tissues by pairwise comparisons of all peptide identification results. The Jaccard index was calculated by dividing the set intersection by the set union for all pairwise comparisons:

J = A n B / A u B
